# Metformin is a Novel Suppressor for Vimentin in Human Gastric Cancer Cell Line

**DOI:** 10.22088/IJMCM.BUMS.10.3.200

**Published:** 2022-01-10

**Authors:** Shiva Valaee, Mehdi Shamsara, Mohammad Mehdi Yaghoobi

**Affiliations:** 1 *Research Department of Biotechnology, Institute of Science and High Technology and Environmental Sciences, Graduate University of Advanced Technology, Kerman, Iran.*; 2 *Department of Animal Biotechnology, National Institute of Genetic Engineering and Biotechnology, Tehran, Iran.*

**Keywords:** Gastric cancer, metformin, vimentin, epithelial-mesenchymal transition

## Abstract

Vimentin, an intermediate filament of mesenchymal cells, is upregulated in epithelial-mesenchymal transition (EMT) and has a main role in cancer metastasis. As a new strategy to control metastatic outgrowth, EMT markers are generally inhibited using some drugs or specific siRNA. In this study, AGS gastric cancer cells were treated with metformin and vimentin-specific siRNA (vim-siRNA) for 48 h. The impact of metformin and vim-siRNA on vimentin downregulation in AGS cells were analyzed by quantitative PCR and Western blot. Following treatment with metformin and vim-siRNA, cell motility, migration and invasion abilities of AGS cells were also analyzed. The results showed that inhibition of vimentin due to metformin was comparable with the vim-siRNA. Furthermore, wound-healing and invasion assays showed a significant decrease in migration and invasion of AGS cells following metformin and vim-siRNA treatment. Our finding for the first time indicated that metformin can be an alternative to specific siRNA for inhibition of vimentin expression and migration of AGS cell line. Taken together, our data indicates that the use of metformin might have a priority to siRNA for inhibition of gastric cancer cell behaviors siRNA is more unstable and expensive than metformin, and needs special vehicles and delivery strategies for efficient transfection of cells. Further *in vivo* studies can reveal metformin's potential in inhibition of EMT and metastasis of cancer cells.

Gastric cancer (GC) is one of the frequently occurring type of malignancy with over one million new cases in 2020 and about 769,000 deaths in both sexes worldwide. Despite a decline in incidence and mortality of GC, it ranks fifth for incidence and fourth for mortality globally (1). 

During embryonic development, epithelial cells acquire mesenchymal phenotype and migrate to tissues. This phenomenon is called Epithelial-mesenchymal transition (EMT). EMT is also a critical step in cancer progress *facilitating* detachment and metastasis of cancer cells. VIMENTIN is a key indicator of EMT which facilitates cell motility and invasion (2). VIMENTIN induction in advanced stages of GC is related to diffuse type of disease, lymphatic invasion, lymph node metastasis and poor prognosis of patients (2, 3). Recently, VIMENTIN has been introduced as an attractive molecular target for diagnostic and therapeutic approaches. Hence, further understanding of drugs leading to reduction of this protein in GC cells can provide new therapeutic approaches to deal with this disease. EMT inhibition and reversion is a crucial step in controlling metastatic outgrowth (4, 5). 

Metformin is a well-known anti-diabetic drug that reduces the incidence of malignancies in patients with diabetes (6). Many studies showed that metformin significantly reduces the occurrence of lung, liver, colon and gastric cancers and related mortality (6-8). In addition to its anti-tumorigenic effects, recent reports on inhibition of EMT genes by metformin support its potential role in fighting cancer metastases. We previously found a significant decrease in cell migration and invasion of GC cell following metformin treatment (9).

Since the discovery of RNAi, siRNA-based therapies have been introduced into clinical trials for treatment of diseases such as cancer (10). siRNA- mediated regression of *VIMENTIN* has been reported in lung cancer by Tadokoro and coworkers. They showed that high expression of VIMENTIN and invasive ability was reduced in non-small cell lung cancer cell lines by knockdown of *VIMENTIN* (11).

In the present study, the impact of metformin and *VIMENTIN*-specific siRNA (vim-siRNA) on VIMENTIN downregulation and cell motility were compared with each other in human gastric adenocarcinoma cell line. We selected AGS cells due to the fact that this cell line is very invasive, and about 95% of GC are caused by adenocarcinoma which has two main types: intestinal and diffuse. Most types of adenocarcinomas are intestinal (12). As the best of our knowledge, this is the first report comparing the outcome of two approaches in suppression of invasion features of GC cells. 

## Materials and methods

**Cell culture**

The AGS cell line was prepared from the National Cell Bank of Iran (Pasture Institute, Tehran, Iran) and cultured in DMEM/F12 medium containing 10% FBS (ThermoFisher Scientific, USA) and 1% penicillin-streptomycin at 37 °C in a CO_2 _incubator.

**MTT test**

The AGS cells were seeded into 96-well plates at a density of 5000 cells/well. The MTT assay was done as we previously published (9). The minimal inhibitory concentration (MIC) of metformin (Santa Cruz, USA) on cell growth was determined 10 mM and chosen for future treatments.

**siRNA Transfection**

siRNAs were purchased from Eurogentec (Belgium). The sequence of vimentin siRNA is ACUUGGAUUUGUACCAUUCdTdT (13). The AGS cells were seeded at 100×10^5^ cells/well in 12 well plates and transfected with 10 pM siRNA. The transfection reagent, Lipofectamine™ 2000 *(Invitrogen, USA),* was used according to the manufacturers’ instruction. To investigate the silencing efficacy of vim-siRNA, the AGS cells were seeded and transfected with 10 pM vim/scrambled-siRNA. Untransfected cells and cells transfected with Lipofectamine™ reagent alone were used as control for transfection. The treated cells were incubated at 37 °C for 48 h before RNA extraction and the medium was replaced 24 h following transfection. 

**Cell migration assay**

The cells were seeded in 6-well plates and cultured till grow as *confluent* monolayer. A line was created by scraping the cells with a sterile pipette in the middle of each well. The cells were then treated with 10 mM metformin or transfected with 10 pM vim-siRNA/scrambled siRNA. The scratched area was marked and cell migration into the scratched area was daily photographed using an inverted microscope equipped with camera (Nikon) for two days.

**Cell invasion assay**

The AGS cells were starved in a serum-free medium one day prior to beginning the assay. Culturex 96 well BME invasion assay kit (R&D systems, USA) was used for invasion assay. Briefly, top chamber was coated with 0.5 X BME solution at 37 ºC in a CO_2_ incubator for 4 h and then plated with 5×10^4^ cells in serum-free DMEM/F12 medium with 10 mM metformin or 10 pM vim/scrambled siRNA. Bottom chamber was filled with medium containing FBS and incubated at 37 ºC with 5% CO_2_ for 24 h. After washing the chambers, diluted Calcein AM stock solution (a reagent in the Culturex kit) was added into the bottom chamber and photographed by fluorescence microscope. The total numbers of invaded cells were counted by Image J software (NIH, version 1.46). All experiments were independently repeated at least three times.

**RNA isolation and reverse transcription**

Total RNA was extracted daily from AGS cells treated with metformin or vim-siRNA for three days using TriPure Isolation Reagent (Roche, Switzerland) according to the manufacture’s instruction. The RNA was subjected to cDNA synthesis using a PrimeScript™ RT reagent kit (Takara, Japan). The reactions volume was 10 µL containing 1 µg extracted RNA, 0.5 µL random hexamers, 0.5 µL oligo-dT and 0.5 µL reverse transcriptase. The tubes were incubated at 37 °C for 15 min and then at 85 °C for 5 s.

**Quantitative polymerase chain reaction (qPCR)**

Real-time qPCR was performed in Rotor-Gene 6000 instrument (Corbett, Australia) using SYBR® Premix Ex Taq^TM^ II kit (Takara, Japan). Thermal cycles were one step 95 ºC for 30 s, 40 cycles of 95 ºC for 5 s and 58 ºC for 30 s. The data were normalized against GAPDH as a reference gene and levels of RNA expression were determined with the 2^−ΔΔCt^ method (14). The sequences of primers were presented in Table 1.

**Western blotting**

Total protein was extracted from controls and metformin/siRNA treated cells by RIPA (radioimmuno-precipitation assay) buffer. The protein content of cell lysate was quantified using Bradford assay (15). Fifty µg cell lysate was loaded and separated on 12% SDS-polyacrylamide gel and transferred onto a polyvinylidene fluoride membrane (PVDF; Roche Diagnostics). The membrane was blocked with 5% BSA in Tris-buffered saline containing 0.1% Tween-20 (TBS-T) for one h at room temperature. The membrane was then incubated with VIMENTIN and GAPDH antibodies (Santa Cruz and Sigma, USA) in TBS-T with 3% BSA for 1 h at room temperature or overnight at 4 ºC. After washing in TBS-T, the membrane was incubated with horseradish peroxidase (HRP)-conjugated secondary antibodies (Abcam, UK) and the proteins were detected using

**Table 1:** The primers sequences used in the qPCR experiments

Name	Accession no.	Sequence (5´ to 3´)	PCR product size
*GAPDH*	NM_001289745	TGGGCTACACTGAGCACCAG	72 bp
		CAGCGTCAAAGGTGGAGGAG
*Vimentin*	NM_003380	CGGGAGAAATTGCAGGAGGAG	106 bp
		CAAGGTCAAGACGTGCCAGAG

Enhanced chemiluminescence reagent (NajmBio Tech, Iran). The antibodies were diluted as 1:10,000 for GAPDH antibody and 1:2000 for VIMENTIN, HRP-linked anti-mouse and anti-rabbit IgG antibodies. The amount of protein level was quantified using Image J software.

**Statistical analysis**

All experiments were repeated three times and the statistical analyses were performed using ANOVA or Student's t-test. A *P*-value <0.05 was considered significant.

## Results

***VIMENTIN*** **level decreased upon treatment with metformin and siRNA**

**Fig. 1:**
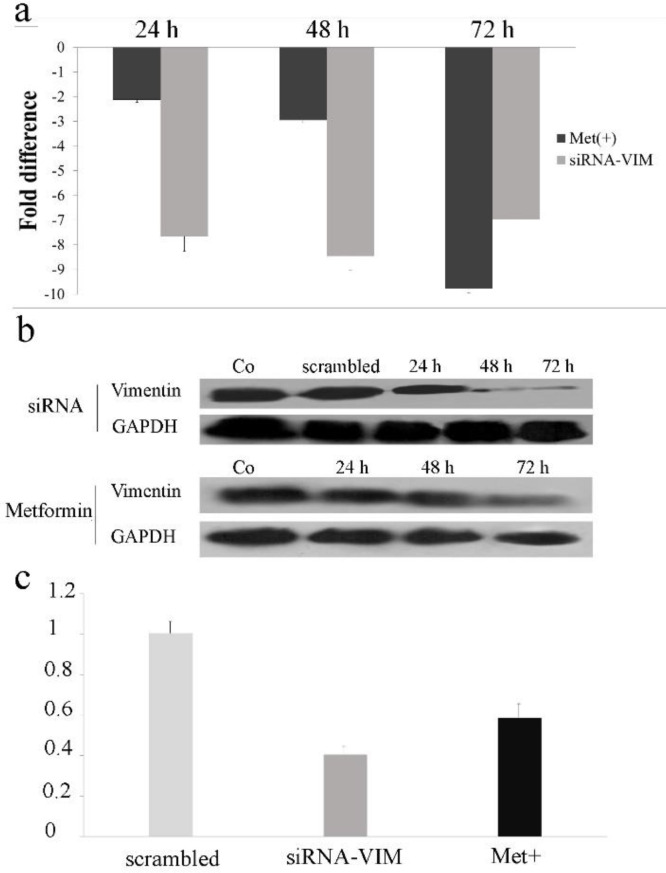
**Quantification of vimentin in 10 mM metformin and siRNA treated cells.** (a) The treated cell was subjected for *vimentin* mRNA level quantification by real-time qPCR for three sequential days. *GAPDH* was amplified in each sample for normalization. Each column shows the mean ± SD of three independent experiments, performed in triplicate. (b) Western blot analysis of vimentin protein. (c) Quantification of vimentin protein in metformin and siRNA received samples. Intensity of the protein bands were quantified using Image J software

*VIMENTIN* expression was measured at mRNA level in metformin and siRNA treated cells by real-time qPCR. Figure 1a shows, 9.78-fold decrease in 10 mM metformin treated cells vs 8.45-fold decrease in siRNA treated cells (P≤ 0.01). What is striking in the figures is that siRNA-mediated downregulation of *VIMENTIN* reaches to maximum level earlier than metformin. While the most reduction of *VIMENTIN* by siRNA was measured at 48 h, it was at 72 h for metformin treated samples.

**Fig. 2:**
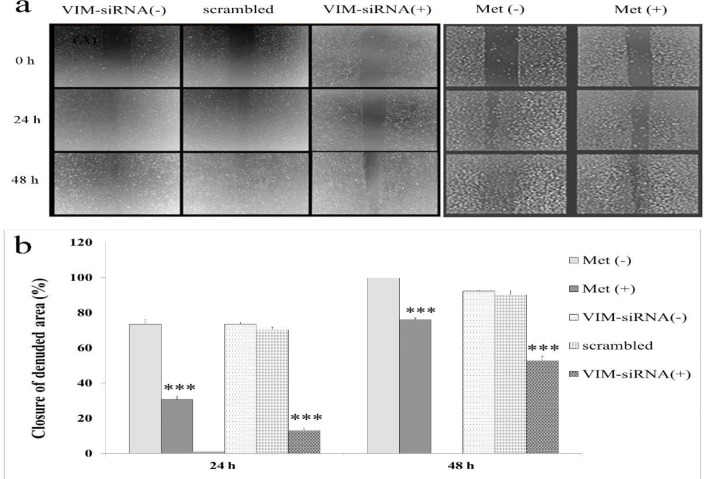
**Effect of metformin on the migration of AGS cells compared with siRNA-VIM treated.** (a) AGS cells were treated with metformin and siRNA for 48 hours (Magnification 40X). (b) After 48 hours in metformin treated 76% and in siRNA treated 56% of scratch was filled. Cell migration was quantified by wound-healing assay using Image J software. ***P≤0.0001

**Fig. 3:**
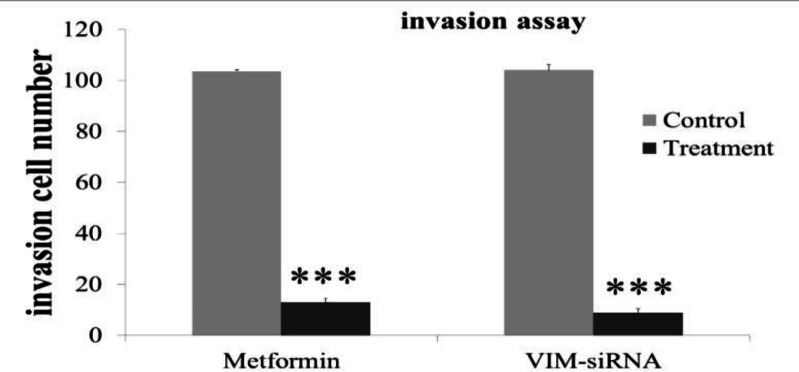
**Effect of metformin on the invasion of AGS cells compared with siRNA-VIM treated.** AGS cells were treated with metformin for 24 hours and the cell invasion was measured in the Culturex 96 well BME invasion assay kit by counting the number of cells invading underside of BME. ***P≤0.0001.

The western blotting results, also confirmed qPCR results. Metformin and siRNA treatments resulted in a significant decrease in VIMENTIN protein level as revealed by western blotting (Figure 1b). VIMENTIN decreased to 41% in metformin treated cells and to 59% in siRNA treated cells following 72 h (P≤0.01) (Figure 1c). 

**Metformin and VIM-siRNA inhibited cell migration and invasion**

Wound healing progress in control culture was rapidly disappeared following 48 h. However, the healing process was inhibited and the closure of denuded area progressed more slowly in metformin and siRNA treatment groups (P≤0.0001) (Figure 2a and b).

The AGS cells plated into transwell chamber, and the number of cell migrated was measured after 24 h. As shown in Figure 3, metformin and siRNA treatment significantly decreased the invasion ability of AGS cells (P≤0.0001). 

## Discussion

 During the last decade, VIMENTIN has received considerable attention regarding its role in cancer cell migration and invasion, signal transduction, and apoptosis. Iwatsuki *et al*. illustrated that circulating VIMENTIN positive GC cells, could reside at bone marrow of GC patients. Also VIMENTIN expression level in bone marrow is clearly related with tumor invasion and lymph node metastasis ability of GC cells (16). Therefore, VIMENTIN is a potential molecular target that can be inhibited to struggle with tumor cells invasion and localization at other tissues.

In this study, we reported high level expression of VIMENTIN in AGS intestinal GC cell line. We also compared the suppressive effects of metformin, as a small drug, and siRNA on VIMENTIN and cell movement. Our results indicated that both molecules potently inhibited VIMENTIN. Nevertheless, the silencing patterns were different, which is probably resulted from different layers targeted by them. Metformin can target mitochondria, interfere with redox systems and some signaling pathways including AMPK and EMT signaling pathway. Metformin leads to reduced expression of transcription factors driving EMT signaling (17, 18). 

Our results indicated the siRNA-induced reduction of *VIMENTIN* was strongly initiated in comparison with metformin. However, metformin emerged as a more potent inhibitor of VIMENTIN following three days. This difference can be described by considering the mechanisms by which siRNA and metformin apply for VIMENTIN suppression. While siRNA directly interacts with *VIMENTIN* transcripts and induces its degradation, VIMENTIN downregulation by metformin is conducted through several known and unknown pathways such as 5'-AMP-activated protein kinase (AMPK) signaling pathway (18). Therefore, the better records were measured for siRNA after 48 h treatment. Increased levels of *VIMENTIN* in day three might be described by intracellular reduction of vim-siRNA, which in turn led to drop off in suppression of *VIMENTIN* by siRNA. However, metformin molecule is more stable than siRNA, as it is not metabolized by the cells. Therefore, it provides a stronger inhibition of VIMENTIN in long-term. 

RNAi-based therapeutics have emerged for treatment of cancers, infectious diseases, and other diseases associated with specific gene disorders (10, 19, 20). However, intracellular stability of metformin is much more than siRNA. Moreover, lower cost of metformin in comparison to siRNA make it more applicable for clinical use.

The changes of VIMENTIN level in response to siRNA and metformin treatments were different from its transcript. While the VIMENTIN protein level in siRNA-treated cells declined intensely than metformin-exposed samples at the day three, the converse results were obtained for *VIMENTIN* transcript amount. This paradox might be described by the difference of half-life between VIMENTIN mRNA and protein. According to previously published study, the half-life of *VIMENTIN* transcript and protein is 6 and 32 h, respectively (21). Therefore, at the third day, transcript of *VIMENTIN* was not detected by qPCR, while a part of earlier expressed proteins was detected by western blot.

In conclusion, our finding for the first time indicated that metformin is able to be an alternative to siRNA for VIMENTIN inhibition in AGS GC cell line. Overall, the use of metformin might have some priority to siRNA. Since siRNA is more unstable and expensive than metformin and it needs special vehicles and delivery strategies for efficient transfection of cells. Further studies in vivo may reveal effectiveness and applicability of metformin in inhibition of metastasis of GC cells. 

## Declaration of Interests

The authors declare that they have no conflict of interest.
